# The *Medicago truncatula* GRAS protein RAD1 supports arbuscular mycorrhiza symbiosis and *Phytophthora palmivora* susceptibility

**DOI:** 10.1093/jxb/erx398

**Published:** 2017-11-25

**Authors:** Thomas Rey, Maxime Bonhomme, Abhishek Chatterjee, Aleksandr Gavrin, Justine Toulotte, Weibing Yang, Olivier André, Christophe Jacquet, Sebastian Schornack

**Affiliations:** 1 University of Cambridge, Sainsbury Laboratory, UK; 2 Laboratoire de Recherche en Sciences Végétales, Université de Toulouse, Centre National de la Recherche Scientifique (CNRS), Université Paul Sabatier (UPS), France

**Keywords:** Arbuscular mycorrhiza, genome-wide association mapping, host susceptibility, *Medicago truncatula*, MtSymSCL3, *Phytophthora palmivora*, RAD1, root colonization, symbiosis, oomycete

## Abstract

The roots of most land plants are colonized by symbiotic arbuscular mycorrhiza (AM) fungi. To facilitate this symbiosis, plant genomes encode a set of genes required for microbial perception and accommodation. However, the extent to which infection by filamentous root pathogens also relies on some of these genes remains an open question. Here, we used genome-wide association mapping to identify genes contributing to colonization of *Medicago truncatula* roots by the pathogenic oomycete *Phytophthora palmivora*. Single-nucleotide polymorphism (SNP) markers most significantly associated with plant colonization response were identified upstream of *RAD1*, which encodes a GRAS transcription regulator first negatively implicated in root nodule symbiosis and recently identified as a positive regulator of AM symbiosis. *RAD1* transcript levels are up-regulated both in response to AM fungus and, to a lower extent, in infected tissues by *P. palmivora* where its expression is restricted to root cortex cells proximal to pathogen hyphae. Reverse genetics showed that reduction of *RAD1* transcript levels as well as a *rad1* mutant are impaired in their full colonization by AM fungi as well as by *P. palmivora*. Thus, the importance of *RAD1* extends beyond symbiotic interactions, suggesting a general involvement in *M. truncatula* microbe-induced root development and interactions with unrelated beneficial and detrimental filamentous microbes.

## Teaser

Using a genome-wide association mapping approach, we identified a new role for the GRAS transcription factor RAD1 in supporting infection by an oomycete root pathogen. Previously, RAD1 was exclusively implicated in symbiosis signalling.

## Introduction

Host compatibility genes facilitate the colonization of plants by pathogenic microbes (van Schie and Takken, 2014). Knowing such host genes and the processes in which they function provides inroads for efforts to establish disease resistance. For example, the knock-out mutant of *MLO* in barley has been successfully deployed to provide resistance against powdery mildew (Acevedo-Garcia *et al.*, 2014). Plant host compatibility genes also function in beneficial root endosymbiosis, such as with arbuscular mycorrhiza (AM) fungi (Gutjahr and Parniske, 2013) and nitrogen-fixing rhizobia (Oldroyd *et al.*, 2011; Popp and Ott, 2011). For instance, commercial wheat varieties often possess a *Reduced height* (*Rht*) dominant allele of *DELLA* suppressing gibberellic acid (GA) signalling, which was recently and unexpectedly found to favour mycorrhiza (Floss *et al.*, 2013). Therefore, breeding for higher yield and reduced height can directly impact AM symbiosis, but may also increase susceptibility to other biotrophic pathogens (Saville *et al.*, 2012). Compatibility genes often control processes essential to general plant biology and physiology, thereby explaining the conservation of those facilitating entry of pathogenic microbial intruders.

Compatibility loci may be a rewarding target to reduce pathogen susceptibility or to support symbiotic associations, but their identification often is challenging. First, genetic detection of susceptibility traits can be masked in pathosystems that are dominated by effective disease resistance responses (Jones and Dangl, 2006; Dangl *et al.*, 2013). Secondly, adapted pathogens may not be fully reliant on basic compatibility genes, since they may specifically reprogram the host to achieve their accommodation *in planta* (Mukhtar *et al.*, 2011). Finally, potential trade-offs between inactivation of host susceptibility genes and implementation of symbiosis-promoting alleles need to be examined (Rey and Schornack, 2013; Evangelisti *et al.*, 2014).

To avoid some of these pitfalls, biotrophic pathogens with broad host range can be used in non-co-evolving pathosystems to uncover susceptibility genes, followed by informed transfer into crops and assessment of crop-relevant pathogens with similar lifestyles (Rey and Schornack, 2013). The oomycete *Phytophthora palmivora* represents a suitable microbe to pursue this strategy, since it infects a number of tropical cash crops but can also colonize *Medicago truncatula*, a model plant for nitrogen-fixing symbiosis and AM symbiosis studies (Evangelisti *et al.*, 2014; Rey *et al.*, 2015). *Phytophthora palmivora* infections start with mobile flagellated zoospores which encyst once settled on targeted root tissues, preferably near the root tip. A germ tube emerges which develops an appressorium on the root epidermis (Wang *et al*, 2012). Entry into the inner root tissues occurs through or between epidermal cells or through wounds. In the root cortex, *P. palmivora* grows largely intercellularly but projects short, specialized hyphae termed haustoria into individual living cortex cells to suppress immunity. This biotrophic stage is followed by necrotrophy when *P. palmivora* kills host tissues through the release of cell wall-degrading enzymes. At this stage, *P. palmivora* can enter the plant vasculature and systemically infect the shoot. Sporangiophores carrying sporangia are formed in the dying tissues, and sporangia serve as propagules or can release several mobile zoospores, thereby completing the asexual life cycle with 2–3 d.

The wealth of genomic (Young *et al.*, 2011), genetic (Pislariu *et al.*, 2012; Urbański *et al.*, 2012), and transcriptomic tools (Benedito *et al.*, 2008; Verdier *et al.*, 2013) available in *M. truncatula* can considerably accelerate molecular characterization of the colonization principles involved in association with *P. palmivora* and possible commonalities with symbiotic processes. Forward genetics of *M. truncatula* identified the dual contribution of a glycerol phosphate acyl transferase gene termed *RAM2* (*Reduced Arbuscular Mycorrhization 2*) in the colonization by AM fungi and the oomycete pathogens *P. palmivora* (Wang *et al.*, 2012) and *Aphanomyces euteiches* (Gobbato *et al.*, 2013). Complementarily, reverse genetics approaches based on surveying mutants defective in symbiosis for their interaction with pathogens revealed that symbiosis-related LysM-RLK receptors, transcription factors, and hormone signalling genes may have broader functions in the associations with microbes (Laffont *et al.*, 2015; Moreau *et al.*, 2013; Rey *et al.*, 2013, 2015, 2016).

As an alternative to a forward-genetics approach, the Medicago HapMap Project (http://www.medicagohapmap.org/) has performed genome resequencing of a core collection of inbred natural accessions of *M. truncatula* to detect single nucleotide polymorphisms (SNPs) at very high resolution, thereby permitting genome-wide association mapping (GWAM) (Atwell *et al.*, 2010; Branca *et al.*, 2011; Stanton-Geddes *et al.*, 2013; Curtin *et al.*, 2017). HapMap accessions have already been used to map the genetic bases of flowering time and traits related to nitrogen fixation (Stanton-Geddes *et al.*, 2013), resistance to osmotic stress (Kang *et al.*, 2015), resistance to the root legume pathogen *Aphanomyces euteiches* (Bonhomme *et al.*, 2014), as well as of the nutritional value of seeds (Le Signor *et al.*, 2017).

Here, we used GWAM on Medicago HapMap accessions to identify new genetic components underlying the response of *M. truncatula* to infection with *P. palmivora*. We measured variation in seedling length upon infection with the oomycete. GWAM revealed a single genomic locus containing two SNPs significantly associated with *P. palmivora*-dependent seedling length, in the promoter region of *MtSymSCL3*, a GRAS transcription factor encoding a gene previously shown to be involved in regulation of root nodule nitrogen-fixing symbiosis (Kim and Nam, 2013). More recently, this locus was termed *Required for Arbuscule Development 1 (RAD1*) and was found to be involved in endomycorrhizal symbiosis of *M. truncatula* and *Lotus japonicus* (Park *et al.*, 2015; Xue *et al.*, 2015). *RAD1* transcript levels were induced during colonization by *P. palmivora* and AM fungi. Transcript knock-down in composite plants and transposon insertion-mediated knock-out in seedlings independently confirmed that *RAD1* is required for *P. palmivora* infection and AM symbiosis. Thus, we demonstrated that *RAD1* is a host susceptibility factor contributing to the colonization of roots by unrelated filamentous symbionts and pathogens.

## Materials and methods

### *Medicago truncatula* HapMap collection and *in vitro* cultivation

*Medicago truncatula* germplasm seeds in the collection of the Medicago HapMap project (http://www.medicagohapmap.org/) were kindly provided by Dr Jean-Marie Prospéri (INRA-Montpellier). A total of 172 lines from the core collection CC192, including the A17 reference line, were phenotyped for *P. palmivora* resistance, whilst 190 lines were used in the uninfected condition. Each accession was phenotyped in three independent repeats with *P. palmivora* and two independent experiments for the uninfected condition. To set up the phenotyping assays, the seeds were gently scratched on sandpaper and left in water for 2 h before being placed on inverted agar plates for 48 h at 20 °C to allow germination. Germinated seedlings were then placed on 1% water–agarose for growth and inoculation with *P. palmivora*. Data for all tested seedlings and mean value for each accession are available in Supplementary dataset S1A–D at *JXB* online.

### *Medicago truncatula* mutant plants

The *M. truncatula* R108 *rad1* transposon insertion mutant NF-9554 (BC1-F4) was obtained from M. Harrison (Boyce Thompson Institute, Ithaca, NY, USA) and has previously been reported (Park *et al.*, 2015).

### *Medicago truncatula* composite plants

To generate composite plants with transgenic roots, A17 seeds were scarified using concentrated sulphuric acid, rinsed three times in water, sterilized with 3% bleach, and rinsed again before soaking for 2 h in sterile water. Seeds were then plated on 2% agar and placed in the dark at 4 °C for 16 h to synchronize their germination, and subsequently transferred to 20 °C in the dark for 24 h before transformation. *Agrobacterium rhizogenes* ARqua1 strain AR1193 (kindly provided by Allan Downie) was electroporated with binary vectors and used to generate composite plants comprising a transgenic hairy root system with non-transformed shoots and leaves (Boisson-Dernier *et al.*, 2001). Transformed plants at 3 or 4 weeks old were selected based on the red fluorescence conferred by the T-DNA insertion, and non-transformed roots were excised prior to further experiments (see ‘Design of constructs‘).

### *P. palmivora* inoculation assays

The strains and cultivation methods used in the current study were previously described by Rey *et al.* (2015). *Phytophthora palmivora* isolates were cultivated on V8 vegetable juice agar plates from which zoospores were released in cold water. The spore concentration was adjusted to 10^5^ spores ml^–1^. For seedling inoculation assays, 10 µl droplets of *P. palmivora* AJ-td or Lili-td carrying a *pTOR::TdTomato* vector (accession nos P6390 and P16830) were placed at the root tip of *M. truncatula* seedlings plated on 0.8% agarose dissolved in ultrapure water. For inoculation of composite plants, transgenic roots were flooded with fluorescently labelled or Lili-YKDEL (accession no. P16830) zoospores carrying a *pTOR::CALYKDEL* construct (kindly provided by Howard Judelson).

### Arbuscular mycorrhizal assays

Arbuscular mycorrhizal assays were conducted in sand substrate as control, which was supplemented with a proprietary preparation of AM fungi provided by Agrauxine in a concentration of 100 propagule units per plant (http://www.agrauxine.com/; France) to assess establishment of AM symbiosis (1:10 w/w). Ten germinated seedlings or composite plants were transferred to pots containing mycorrhizal substrate. Plants were grown for 6 weeks in glasshouse conditions (day temperature 26 °C, night 19 °C, 65% humidity, and 16 h of 165 W m^–2^ light) and watered every 2 d with Long-Ashton nutrient solution (Balzergue *et al.*, 2011). The fertilizer phosphate concentration was 0.37 g NaH_2_PO_4_×H_2_O per litre. At the end of the assay, seedlings were suspended in water to separate them and remove sand. For composite plants, newly formed untransformed roots were removed prior to scoring of the extent of mycorrhizal colonization based on absence of a DsRed fluorescent signal in their tissues (see ‘Design of constructs’). Assessment of mycorrhizal colonization was performed on each plant both microscopically (Balzergue *et al.*, 2011) and by quantification (see ‘Gene expression assays’) of *M. truncatula* AM markers *MtBCP1* and *MtPT4* (Lauressergues *et al.*, 2012).

### Phenotype modelling and association mapping

SNP data were available for 160 lines used in the *P. palmivora* dataset and 175 lines used in the non-infected dataset. We calculated adjusted means of root length for each *M. truncatula* accession by fitting a mixed linear model in which accessions had fixed effects and the biological repeat and boxes were considered as random effects. GWAM was performed on these adjusted means with the compressed mixed linear model approach and a Q+K model accounting for population structure and kinship among *M. truncatula* accessions in the core collection (Yu *et al.*, 2006; Kang *et al.*, 2010; Zhang *et al.*, 2010), implemented in the software TASSEL (Bradbury *et al.*, 2007), with parameters described in Bonhomme *et al.* (2014). A set of 5 329 189 SNPs with minor allele frequency (MAF) ≥0.05 with maximum 10% missing counts was used for GWAM. To identify significant associations, we applied a genome-wide 5% significance threshold with Bonferroni correction for the number of linkage disequilibrium (LD) blocks in the genome, leading to a significance threshold of 10^–6^ for the *P*-value, following Bonhomme *et al.* (2014).

### Gene expression assays

RNA extraction was performed with 100 mg of pooled plant material for each biological replicate (RNeasy Plant Minikit, QIAGEN), reverse transcription of first-strand cDNA (Transcriptor First Strand cDNA Synthesis Kit, Roche), and quantitative PCR (qPCR) analyses were performed and analysed as described in Rey *et al.* (2015). In brief, *M. truncatula* stable transcripts *MtH3l*, *MtUBQ*, and *MtTefa* were used to standardize expression of *M. truncatula RAD1* and defence genes of interest (Mtr.9569.1.S1_at, Medtr4g083710, and Medtr1g075340) in seedlings and hairy roots (Nars *et al.*, 2013). In addition, quantitative reverse transcription–PCR (RT–qPCR) was also use to examine accumulation of *P. palmivora* biomass by measuring expression of the oomycete housekeeping genes *Elongation factor 1α* (*Ef1α*) and *ribosomal protein S3a* (*WS21*), which were identified by homology to *Phytophthora parasitica* transcripts (Yan and Liou, 2006). Expression of the *in planta* induced *P. palmivora* effector gene *REX3* (Evangelisti *et al.*, 2017) was normalized to *WS21* to track its biotrophy-stage induction. The 2^−ΔCp^ and 2^−ΔΔCp^ methods were used to display gene expression levels (Livak and Schmittgen, 2001). Primers used in this study are listed in Supplementary Table S1.

### Design of constructs

The 3′-untranslated region (UTR) of *RAD1* (Medtr4g104020) was identified in the *M. truncatula* v 4.0 genome release and supported by RNAseq coverage (http://jcvi.org/medicago/). A 177 bp fragment of the 3'UTR was amplified from *M. truncatula* A17 cDNA using Phusion DNA polymerase (New England Biolab Inc., UK) with primers listed in Supplementary Table S1. This sequence was unique in the *Medicago* genome and did not return any blast hit other than the query. Amplicons were immediately ligated into the pENTR/D-TOPO Cloning Kit (Invitrogen) used as an entry Gateway vector, and transformations were carried out into TOP10 chemically competent *Escherichia coli* (Invitrogen). Minipreps were carried out using a QIAprep Spin Miniprep Kit (QIAGEN). Then recombination was performed into a pK7GWIWG2_II-Red Root vector (VIB, Ghent University, Belgium) using Gateway LR Clonase II Enzyme Mix (Invitrogen) to produce *hpRAD1* binary constructs. A control recombination with the pENTR-GUS clone provided with the LR kit was performed, and the resulting vector was used as a control in the experiment and designated *hpuidA*.

### Microscopy

Imaging of *P. palmivora* colonization in infected root sections was performed on excised root tissues mounted in water and covered by coverslips. Using a Leica TCS SP8 confocal microscope equipped with HyD detectors, the *P. palmivora* AJ-td fluorescence was visualized with the 561 nm laser for excitation and the HyD detector detecting fluorescence in a 570–600 nm window.

Quantification of *P. palmivora* colonization in R108 (and the corresponding *rad1* mutant background) was performed on confocal images of infected seedlings at 24 h after inoculation. Confocal images were obtained at the same developmental area of each seedling radicle (2–3 mm from the radicle tip). After selecting the saturation channel, background subtraction was performed. Then, the area of pathogen was selected by creating a binary image, with black pixels representing the *P. palmivora* signal distribution and white pixels representing non-colonized tissue. The area of black pixels in the binary images was then quantified using ImageJ/Fiji to determine the extent of pathogen spread.

For the imaging of transgenic hairy roots, we used the *P. palmivora* Lili-YKDEL strain to avoid interference with the DsRed fluorescence expressed by transgenic roots. Hence, the yellow fluorescent protein (YFP) signal was detected using 514 nm excitation and 520–550 nm emission, and DsRed was detected using 561 nm excitation and 570–600 nm emission. Images represent stacks of 20–25 slices of 1 μm in maximum-intensity projections merged with inverted transillumination images to outline cells. Line averaging of 4 was applied to reduce the noise-to-signal ratio. Pictures were processed with ImageJ software v1.46 including Fiji plug-ins. Complementary light microscopy imaging was carried out using a Leica M165 FC fluorescence stereomicroscope equipped with a DFC310FX camera. The DSR filter (10447412) was used to detect the tdTomato produced by *P. palmivora* AJ-td. The same settings were used to screen for monomeric DsRed-expressing hairy roots of *M. truncatula* composite plants. For mycorrhiza assays, the percentage mycorrhization was established using the grid intersect method as described in Lauressergues *et al.* (2012). Root samples were boiled in 10% KOH for 7 min, washed three times in water, and ink-stained at 95 °C for 3 min (Scheaffer ink, 5% solution) before being rinsed in 0.5% acetic acid and then mounted between slides and coverslips. SEM was performed in variable pressure mode using Zeiss EVO HD15.RNA *in situ* hybridizations of root sections.

### Probe synthesis and histochemical detection of transcripts

To generate gene-specific probes, cDNA fragments were amplified using primers 5'-CACCATGTCACCTGCACTTTATGCTAGTACC-3' and 5'-GCATTTCCAGCAAGAAA CTGCAACAATAGG-3' for *RAD1*, and 5'-ATGGTGAGCAAGGGCGAG-3' and 5'-CTTGTACAGC TCGTCCAT-3' for *GFP* (green fluorescent protein). The fragments were ligated into the pGEM-T Easy vector (Promega) and verified by sequencing. The constructs were then used as templates for *in vitro* transcription using the DIG RNA Labeling Kit (Roche). *Medicago truncatula* A17 roots were fixed in FAA (formaldehyde, acetic acid, ethanol), embedded in wax, and cut into 8 μm sections. The sections were processed according to http://www.its.caltech.edu/~plantlab/protocols/insitu.pdf and hybridized with *RAD1* or *GFP* probes. After incubation with anti-digoxigenin antibody (Roche), the signals were detected by NBT/BCIP colour reaction (Roche).

### Accession numbers

Sequence data referred to in this article can be found in the EMBL/GenBank databases under the following accession numbers: *MtSymSCL3/RAD1* (MTR_4g104020), *MtTefa* (MTR_6g021800), *MtUBQ* (MTR_3g091400), *MtBCP1* (MTR_7g086190), *MtPT4* (MTR_1g028600), *Thaumatin-PR5* (Mtr.9569.1.S1_at), *Anionic-Peroxidase* (MTR_4g083710), and *Protease-Inhibitor* (MTR_1g075340).

## Results

### Genome-wide association mapping of infected seedling length identifies *RAD1*

**Fig. 1.**
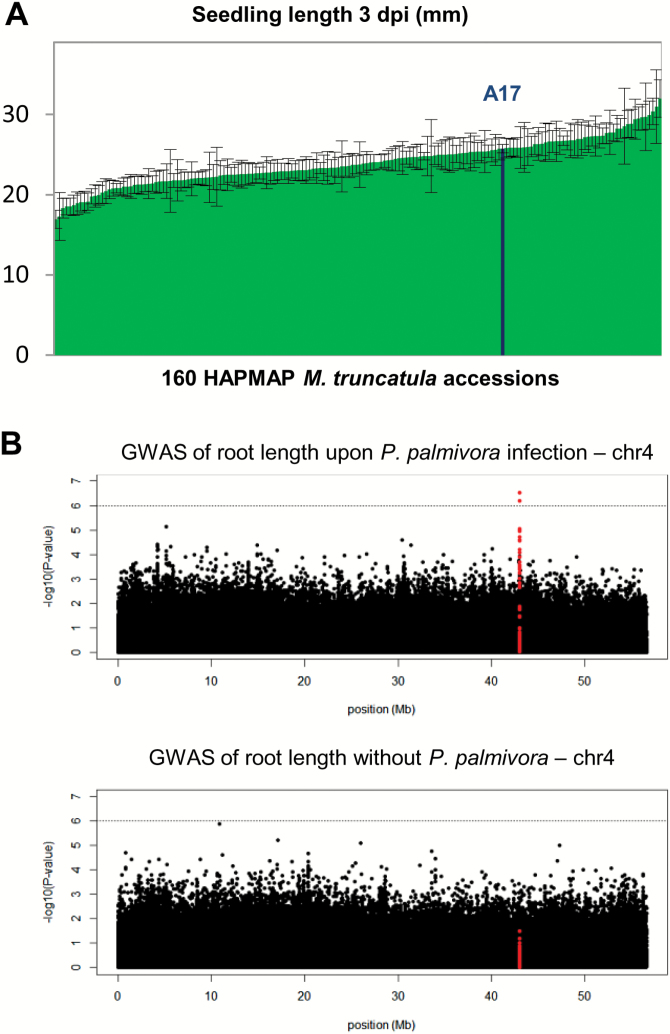
Variation of length of seedlings infected with *P. palmivora* across the HapMap collection of *M. truncatula* accessions revealed a contribution of Medtr4g104020. (A) Variation in seedling length across all tested accessions at 3 days post-infection (dpi) with *P. palmivora* AJ-td. (B) Genome-wide association mapping identified a unique genomic region significantly associated (*P*<10^–6^) with seedling length upon infection of *M. truncatula* with *P. palmivora*. Manhattan plot of SNP positions on chromosome 4 and their significance values [–log10(*P*)] shows two candidate SNPs positioned upstream of Medtr4g104020 (SNPs in red cover 5 kb upstream plus Medtr4g104020 gene model). (This figure is available in colour at *JXB* online.)

To assess the diversity of *M. truncatula* responses to root colonization by *P. palmivora*, we surveyed the collection of natural *M. truncatula* accessions sequenced by the Medicago HapMap project. In total, germinated seedlings of 172 lines, among which 160 had SNP data available (on average 13 seedlings per line), were infected with *P. palmivora* AJ-td zoospores (Supplementary dataset S1A, B). We noticed a striking variation in seedling length of the infected HapMap lines ranging from 16 mm to 32 mm at 3 days post-infection (dpi) (Fig. 1A), when first root rot symptoms can be observed, due to the shift between biotrophic and necrotrophic stages in the pathogen’s life cycle (Rey *et al.*, 2015) (Supplementary Fig. S1).

To investigate the genetic components contributing to this variation in seedling length, a GWAM was carried out using genotypic data at 5 329 189 SNPs on 160 lines spanning the range of *M. truncatula* diversity. These SNPs, characterized by MAF ≥0.05 and maximum 10% missing counts, were analysed in a mixed linear model (Q and K matrix) to reduce false positives generated by population structure and kinship among accessions (for further details, see the Materials and methods).

Narrow-sense heritability was estimated at 0.35 for seedling length upon infection in the *M. truncatula* collection, and we detected a single locus in the last third of chromosome 4 (Fig. 1B) with two significant SNPs (*P*≤10^–6^) located at positions –4251 and –2467 upstream of the predicted ATG codon of *RAD1*, a GRAS (GAI, RGA, SCR) transcription factor-encoding gene (Medtr4g104020) (Table 1; Supplementary Fig. S2; Supplementary dataset S1E). LD analysis of the region encompassing the two SNPs showed that Medtr4g104020 is the only gene within an LD block of ~20.6 kb (Supplementary Fig. S2), suggesting that it is a strong candidate for the observed seedling length variation upon infection. Conversely, a similar GWAM on seedling length of 175 uninfected accessions (Supplementary dataset S1C, D) did not detect any significant SNPs associated with seedling length in this region (Fig. 1B). In addition, no significant SNPs reaching the –log10^–6^ threshold could be detected in a genome-wide analysis of uninfected seedling length (Supplementary Fig. S3). This suggests that natural variation linked to *RAD1* is specifically implicated in infected seedling responses and that *RAD1* is the major regulator of seedling length variation in the conditions of our screening.

**Table 1.** Candidate SNPs (Mt4 genome version) associated with extent of symptoms and length of seedlings upon *P. palmivora* inoculation

Chromosome	Position	*P*-value	Reference allele	SNP	Gene region	Gene model	Annotation
4	43024916	6.5 × 10^−7^	C	T	Non-coding	Medtr4g104020	*MtSymSCL3/RAD1*
4	43026700	3.03 × 10^−7^	T	C	Non-coding	Medtr4g104020	*MtSymSCL3/RAD1*

### *RAD1* is expressed locally during *P. palmivora* root cortex colonization

**Fig. 2.**
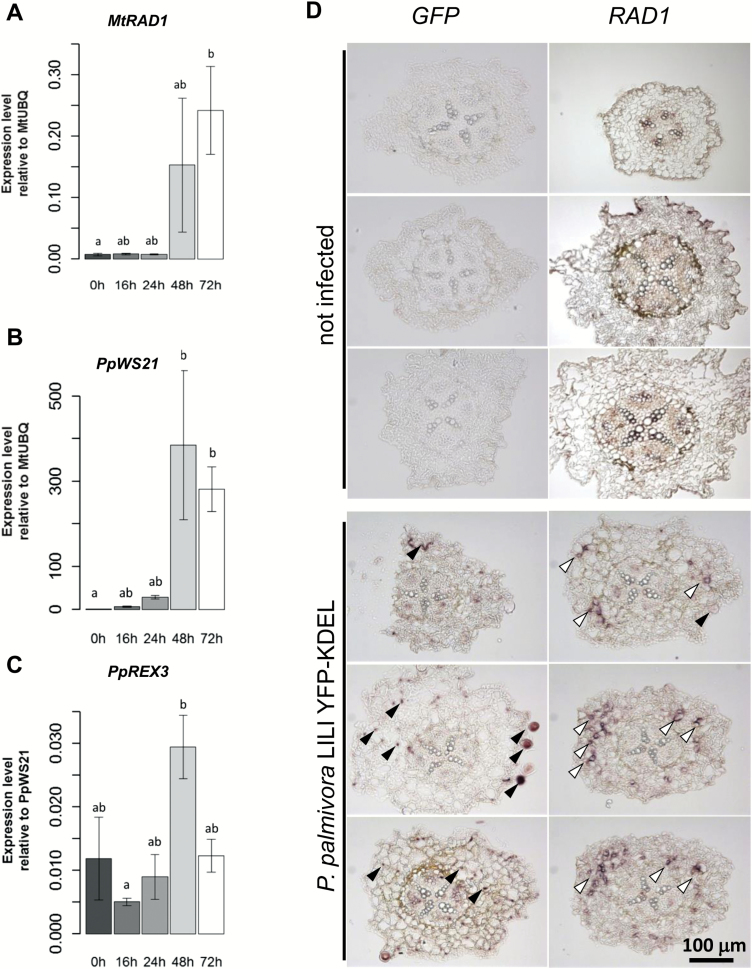
Expression of *MtRAD1* is locally induced during cortex infection with *P. palmivora*. (A–C) Expression levels of *P. palmivora WS21* (a), *M. truncatula RAD1* (b), and the *P. palmivora REX3* effector candidate gene (c) at different hours post-infection (four replicates). Expression levels are calculated relative to *MtUBQ* (a, b) and relative to *PpWS21* (c). Bars and errors bars indicate means ±SD of *n*=4. Comparisons made using Kruskal–Wallis and Nemenyi’s test of multiple comparisons for independent samples (Tukey). Means with different group letters are significantly different (*P*<0.05) for (a) and (c). and significantly different (*P*<0.01) for (b). (D) *In situ* hybridization using *GFP* or *RAD1* probes on uninfected and infected *M. truncatula* A17 seedling root sections. *GFP* probes label expression of *YFP-KDEL* inside hyphae and sporangia of *P. palmivora* (black arrows). The *RAD1* probe labels localized expression within cortex cells (white arrows).

We assessed *RAD1* expression levels during a time course of infection with *P. palmivora* LILI-YKDEL, which constitutively expresses an endoplasmic reticulum-targeted YFP, allowing for *in vivo* imaging of infection structures. *RAD1* expression levels increased 48 h post-inoculation (hpi; Fig. 2A) when the *PpWS21* housekeeping gene expression started to accumulate in host tissues (Fig. 2B), during the biotrophic infection stage where *P. palmivora* colonized the cortex (Supplementary Fig. S1). Furthermore, *RAD1* expression induction coincided with peak expression levels of the RXLR effector gene *REX3* (Evangelisti *et al.*, 2017) (Fig. 2C) and the occurrence of haustoria (Rey *et al.*, 2015), both indicators for the biotrophic stage of infection. We carried out *in situ* hybridization of *M. truncatula* A17 seedlings under mock conditions or upon biotrophic colonization by *P. palmivora*. Wax-embedded seedlings were cut and hybridized with a *GFP* probe detecting YFP-expressing hyphae of *P. palmivora* LILI-YKDEL in the root cortex as well as sporangia formed outside the root (Fig. 2D). When we hybridized similar sections from the same samples with a full-length *RAD1* probe encompassing the ORF, we found localized hybridization signals resembling the pattern of *P. palmivora* colonization, whereas the majority of the root tissues did not show any hybridization (Fig. 2D). No strong *RAD1* probe signal was observed in uninfected seedlings. From these data, we conclude that *RAD1* expression is induced during biotrophic colonization by *P. palmivora* and restricted to a few cells.

### Knock-down of *RAD1* impairs colonization by arbuscular mycorrhiza fungi

To assess a requirement for *RAD1* in microbial colonization, we devised a hairpin silencing construct (*hpRAD1*) specifically targeting only the single-copy *M. truncatula* A17 *RAD1* gene. Nucleotide BLAST (basic local alignment sequence tool) searches against the *M. truncatula* genome Mt4.0v1 did not identify any other sequences with significant similarity, making off-target effects highly unlikely.

To confirm functional knock-down of *RAD1*, we transformed roots of *M. truncatula* A17 with either *hpRAD1* or an *hpuidA* control construct using *Agrobacterium rhizogenes*-mediated transformation (Boisson-Dernier *et al.*, 2001) and tested for a reduction in AM fungus colonization which was recently reported (Park *et al.*, 2015). A constitutively co-expressed DsRed encoded by the same T-DNA served as a red fluorescent cellular marker to identify transgenic roots. We did not observe significant macroscopic alterations of root system development upon transformation (Supplementary Fig. S4).

**Fig. 3.**
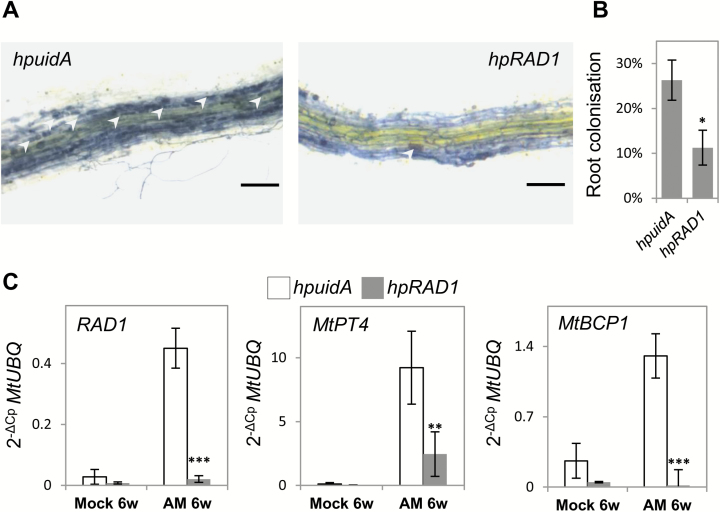
Expression of *hpRAD1* reduces *RAD1* transcript levels as well as the degree of mycorrhization by mixed arbuscular mycorrhiza (AM). (A) Ink staining of mycorrhizal structures in *hpuidA* and *hpRAD1* hairy roots (scale bars=200 µm); white arrowheads indicate mycorrhizal arbuscules; brightness has been enhanced in both images to increase visibility of arbuscule-filled cells. (B) Quantification of the overall degree of AM fungal colonization within root systems expressing hairpin silencing constructs targeting *uidA* or *RAD1*. (C) Transcript levels of *RAD1* and mycorrhizal symbiosis markers *MtPT4* and *MtBCP1* in roots expressing *hpuidA* and *hpRAD1* constructs and grown in control conditions (*n*=3) or in AM fungi mixed inoculum (*n*=4). Each sample consists of five composites plants comprising at least four transformed roots, Student’s *t*-test was applied between constructs in each condition to compare standardized gene expression using *MtUBQ* as housekeeping gene and the 2^−ΔCp^ method (**P<0.01, ***P<0.001). Error bars show the SE.

We grew composite plants carrying *hpRAD1* or *hpuidA* for 6 weeks in a sand substrate with or without added AM fungi inoculum. After 6 weeks, transformed roots of each composite plant were subjected to ink staining to detect intraradical fungal structures. RNA extraction was carried out to validate the efficiency of *RAD1* silencing and to quantify transcriptional induction of *M. truncatula* AM symbiosis marker genes. Using the grid intersect method (Balzergue *et al.*, 2011), we detected 26% mycorrhizal colonization in *hpuidA* control roots versus 11% in *hpRAD1* (Fig. 3A, B). Visual inspection of mycorrhizal structures showed a lower amount of arbuscules in *hpRAD1* (Fig. 3A) at the time of harvest. RT–qPCR analysis validated a 17-fold transcriptional induction of *RAD1* in AM fungus-colonized roots carrying the *hpuidA* control construct, and this transcriptional induction was abolished in *hpRAD1* roots (Fig. 3C). To assess the amount of arbuscules formed in *hpuidA* and *hpRAD1* roots, we quantified arbuscule-specific gene expression of the phosphate transporter *MtPT4* and the blue copper-binding protein *MtBCP1* (Lauressergues *et al.*, 2012). While these genes are respectively up-regulated 76 and 5 times in *hpuidA*-expressing mycorrhized roots, we detected significantly lower transcript levels in *hpRAD1*-expressing mycorrhized roots (Fig. 3C). Thus, our RT–qPCR and visual assessment of the extent of mycorrhizal colonization in *hpRAD1* roots confirmed a role for *RAD1* in AM fungus colonization and also the functionality of our hairpin knock-down construct.

### *RAD1* is required for full *P. palmivora* colonization

**Fig. 4.**
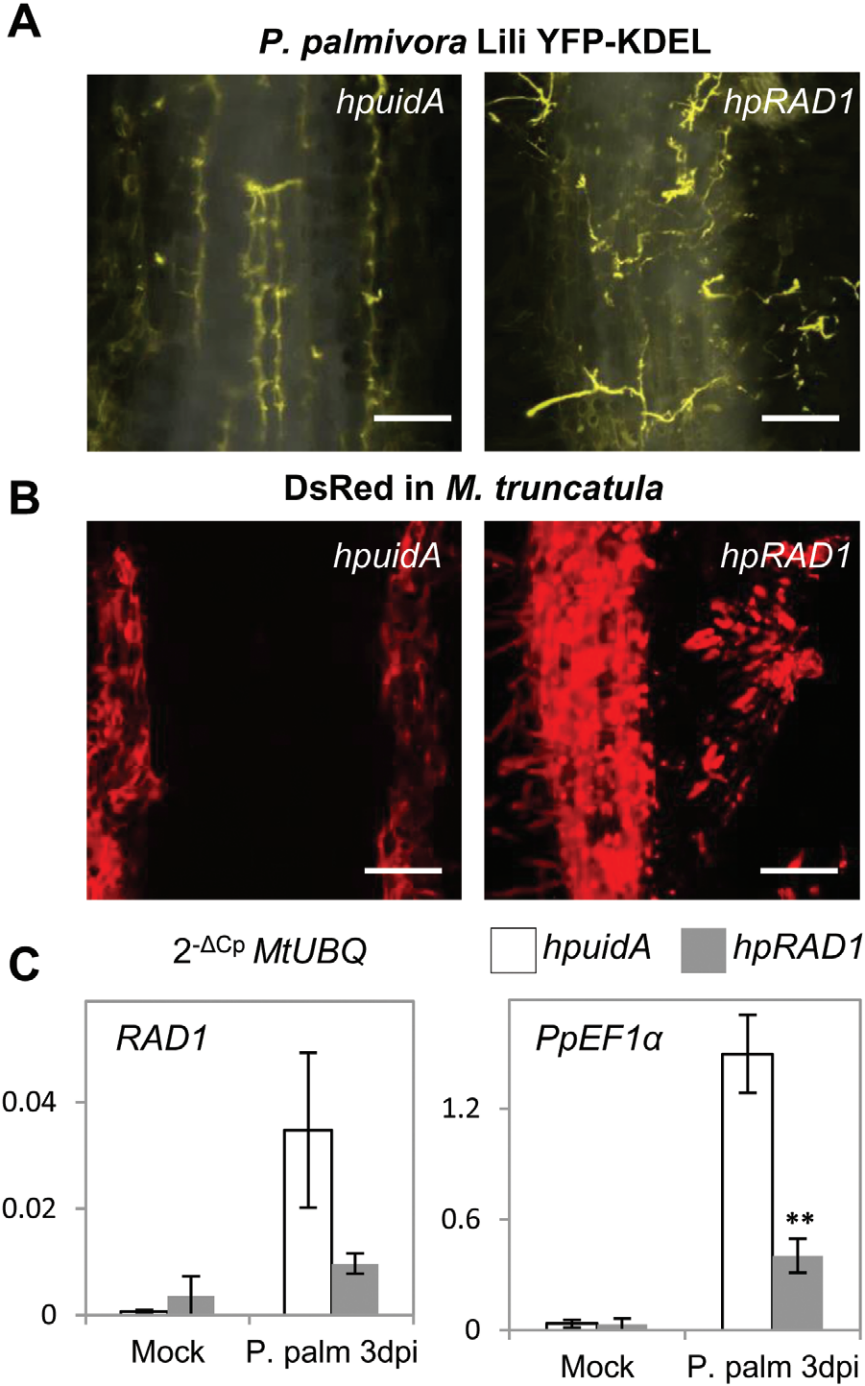
*Medicago truncatula* A17 roots expressing *hpRAD1* silencing constructs are impaired in colonization by *P. palmivora* Lili-YKDEL. (A) Overlay of maximum projections of inverted transmitted light from rhizodermis and *P. palmivora* Lili-YKDEL yellow fluorescence (scale bars=200 µm). (B) Maximum projection of red fluorescence from expression of nucleocytoplasmic DsRED (scale bars=200 µm). (C) Transcript levels of *RAD1* and *PpEF1α* in roots expressing *hpuidA* and *hpRAD1* constructs grown in control conditions (white bars, *n*=3) or upon infection with *P. palmivora* Lili-YKDEL (grey bars, *n*=4). Student’s *t*-test was applied to compare gene expression between constructs in each condition using *MtUBQ* as housekeeping gene and the 2^−ΔCp^ method (**P<0.01). Error bars show the SE.

To decipher the relevance of *RAD1* in the interaction with *P. palmivora*, we infected 6-week-old *M. truncatula* A17 roots expressing *hpuidA* or *hpRAD1* with *P. palmivora* Lili-YKDEL (Chaparro-Garcia *et al.*, 2011; Rey *et al.*, 2015) spores and carried out visual, microscopic, and molecular analysis of the colonization. At 3 dpi, oomycete hyphae were spreading in cortical tissues along the *hpuidA* roots (Fig. 4A). Colonization was associated with a decrease in red fluorescence from *DsRed*-co-expressing cells harbouring the hairpin *uidA* constructs, indicating the start of disintegration of infected tissues (Fig. 4B). In contrast, hyphae were detected mostly at the surface of *hpRAD1* roots, and the underlying tissues remained red fluorescent and viable (Fig. 4A, B). We quantified the expression of *RAD1* in these roots and observed 47 times higher steady-state transcript levels in *hpuidA* roots infected with *P. palmivora* at 3 dpi compared with uninfected roots (*P*=0.09; Fig. 4C). By comparison, *RAD1* was very weakly induced in *hpRAD1*-expressing roots (*P*=0.3), reaching only 27% of the expression level detected in colonized *hpuidA* roots (*P*=0.09; Fig. 4C). Therefore, we concluded that the *hpRAD1* construct successfully restricted the *P. palmivora*-triggered up-regulation of *RAD1* transcript levels, preventing a full transcriptional response. To quantify *P. palmivora* in these roots, we then measured the expression of *P. palmivora Ef1α* as a biomass marker and observed 75% lower levels in *hpRAD1* roots versus *hpuidA* (*P*=0.008; Fig. 4C). Hence, full *RAD1* transcript induction is required for optimal establishment of *P. palmivora* colonization. Activation of genes implicated in defence was not significantly higher in plants expressing *hpRAD1*, suggesting that the observed reduction in pathogen biomass in these lines is not attributable to a stronger defence response (Supplementary Fig. S5).

**Fig. 5.**
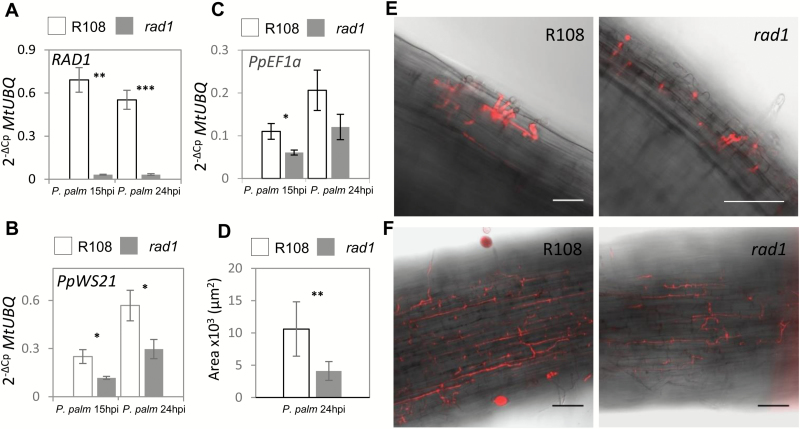
The *M. truncatula rad1* mutant is impaired in colonization by *P. palmivora.* (A) Transcript levels of *RAD1* in R108 (white bars) and *rad1* (grey bars) upon infection with *P. palmivora* Lili-Td. (B) Transcript levels of *PpEF1α* in R108 (white bars) and *rad1* (grey bars) upon infection with *P. palmivora* Lili-Td. (C) Transcript levels of *PpWS21* in R108 (white bars) and *rad1* (grey bars) upon infection with *P. palmivora* Lili-Td. For each data point, *n*=4 biological replicates were analysed. Student’s *t*-test was applied to compare gene expression between lines in each condition using *MtUBQ* as housekeeping gene and the 2^−ΔCp^ method. (D) *P. palmivora* progress at 24 hpi in seedling roots of R108 (*n*=13) and *rad1* (*n*=15) measured as surface area after binary conversion (Fiji) of confocal images. (E) Confocal microscopy of *M. truncatula* R108 and the *rad1* mutant 5 hpi with *P. palmivora* Lili-Td. (F) Confocal microscopy of *M. truncatula* R108 and the *rad1* mutant 24 hpi with *P. palmivora* Lili-Td.(*P<0.05; **P<0.01; ***P<0.001). Error bars show the SD. Scale bars=30 µm.

We furthermore assessed *P. palmivora* accumulation in infected *M. truncatula rad1* mutant seedlings (Park *et al.*, 2015) and their corresponding *M. truncatula* R108 wild-type genotype. Transposon-insertion *rad1* lines displayed strongly reduced *rad1* transcript levels compared with the wild type (Fig. 5A). Quantification of *P. palmivora WS21* or *EF1α* transcript levels was carried out at 15 hpi and 24 hpi as the infection progresses much faster in *M. truncatula* R108 compared with A17 (Huisman *et al.*, 2015). We found a significant reduction in *P. palmivora* biomass in *rad1* mutants compared with the wild type at both time points (Fig. 5B, C). At 24 hpi, we observed significantly larger amounts of *P. palmivora* hyphae in infected wild-type R108 roots as compared with *rad1* roots (Fig. 5D, F). At an earlier time point of 5 hpi when *P. palmivora* had just crossed the epidermal barrier, there was no clear difference between the mutant and wild type (Fig. 5E). This suggests that *rad1* insertion lines are impaired in root cortex colonization, matching the observed cortex-based expression of *RAD1* (Fig. 2). In summary, *RAD1* contributes to *P. palmivora* colonization processes in the *M. truncatula* accessions A17 and R108.

## Discussion

We used GWAM to identify genes contributing to seedling length upon root colonization of *M. truncatula* by the broad host range hemibiotrophic oomycete pathogen *P. palmivora*. *Medicago truncatula* is a model system for root–microbe interactions with a strong emphasis on symbiotic associations formed with AM fungi and nitrogen-fixing bacteria (Boisson-Dernier *et al.*, 2001). In contrast to the AM symbiosis-incapable GWAM workhorse Arabidopsis (Atwell *et al.*, 2010), *M. truncatula* enables cross-checking the role of compatibility genes identified for either AM symbiosis or pathogen colonization without the need to identify orthologous genes of the other species (Evangelisti *et al.*, 2014; Rey and Schornack, 2013).

Here, we identified two SNPs upstream of *RAD1* as significantly correlated with the length of *P. palmivora*-infected seedlings, but not with seedling length in uninfected conditions. Notably, *RAD1* was not identified as a candidate in a GWAM study of another *Medicago* root-infecting oomycete, *Aphanomyces euteiches* (Bonhomme *et al.*, 2014). This may suggest that *RAD1* contemporary evolution contributes differentially to different root infections, and further experiments are required to test this hypothesis. Attempts to use GWAM in the detection of genetic loci under natural variation in AM have been unsuccessful (Dreher *et al.*, 2017); therefore, the use of a biotrophic pathogen such as *P. palmivora* to identify common genetic traits underlying root colonization may provide inroads to identifying novel symbiosis-relevant genes.

Mutants in *RAD1* have reduced, but not abolished AM fungal colonization (Park *et al.*, 2015). Furthermore, hairpin silencing of *RAD1*, then named *MtSymSCL3*, resulted in higher nodule numbers (Kim and Nam, 2013), implicating *RAD1* in two different symbiotic plant–microbe interactions and lending further support to its dual role in root development and plant–microbe interactions. In contrast to the pathogen-inducible *RAD1*, the constitutively expressed *L. japonicus* GRAS transcription factor gene *NSP2* contributes to seedling root length in uninfected scenarios (Murakami *et al.*, 2013). *RAD1* transcript accumulation during *P. palmivora* infection was spatially restricted and similar in distribution to the root cortex-colonizing biotrophic hyphae. Furthermore, the highest *RAD1* expression levels during infection coincided with those of the RXLR effector gene *REX3*. *REX3* and other RXLR effector genes have been demonstrated previously to have peak expression levels during biotrophy (Haas *et al.*, 2009; Evangelisti *et al.*, 2017) and can therefore be used as markers for this developmental stage of infection. Thus, it is likely that *RAD1* expression is limited to the root cortex cell in the vicinity of biotrophic *P. palmivora* hyphae, similar to the observed expression pattern of *LjRAD1* promoter–GUS (β-glucuronidase) reporters in arbuscule-containing tissues (Park *et al.*, 2015; Xue *et al.*, 2015).

To study the requirement for *RAD1* to achieve a full *P. palmivora* root infection, we employed both a silencing approach in the previously established *M. truncatula* A17–*P. palmivora* pathosystem (Rey *et al.*, 2015) and a recently described transposon-insertion line in seedlings of the accession R108 background. Knock-down of *RAD1* transcripts in *M. truncatula* A17 significantly reduced *P. palmivora* infection as well as AM fungal colonization and arbuscule formation, and the *rad1* mutant has previously been characterized as being impaired in AM fungal symbiosis (Park *et al.*, 2015). Importantly, we demonstrate a role for *RAD1* in both infections of fully developed root systems of composite plants and seedling root infections. Therefore, *RAD1* is required for full colonization by a filamentous pathogen as well as the unrelated beneficial AM fungi. RAD1 is unlikely to function as a negative regulator of defence since we did not observe higher defence gene expression in roots expressing *hpRAD1* constructs which displayed reduced pathogen biomass (Supplementary Fig. S5).

The evolutionary history of *RAD1* predicts a conservation for AM fungal symbiosis (Bravo *et al.*, 2017). Our results demonstrate that plant genes which were hypothesized to be specifically involved in AM formation can also be exploited by pathogenic filamentous microbes, as previously demonstrated (Wang *et al.*, 2012). It will be interesting to test further genes whose presence is evolutionarily linked with AM symbiosis for their role in plant–pathogen interactions to unravel the extent of overlap between both types of interaction outcomes.

*RAD1* has previously also been implicated in the formation of intracellular arbuscules by AM fungi in *L. japonicus* roots (Park *et al.*, 2015; Xue *et al.*, 2015). We did not observe haustorium formation in *hpRAD1*-expressing hairy roots, but this is likely to be attributable to an overall lack of intraradical colonization. Thus, a potential role for *RAD1* in haustorium formation remains to be addressed.

GRAS proteins are transcription regulators and form homo- and heterodimers to fulfil their function (Hirsch *et al.*, 2009; Gobbato *et al.*, 2013). RAD1 has been shown to interact with the symbiosis-associated GRAS proteins NSP1, NSP2 (Nod Signaling Pathway 1,2), RAM1 (Required for Arbuscular Mycorrhiza 1), and the GA signalling component DELLA (Park *et al.*, 2015; Xue *et al.*, 2015; Floss *et al.*, 2016). Therefore, this RAD1 associated network of GRAS proteins can integrate microbe-derived signals such as lipo-chitooligosaccharides or short chitooligosaccharides (Oldroyd, 2013) as well as endogenous hormonal cues (Floss *et al.*, 2016; Pimprikar *et al.*, 2016). RAD1-containing GRAS complexes shape the root system, and symbiotic and immune responses, thereby controlling the outcome of interactions with beneficial and detrimental microbes, and it is therefore not unexpected that our GWAM of infected seedling lengths picked up *RAD1* as a candidate gene. Nonetheless, the array of downstream targets of *RAD1* in the control of seedling elongation upon *P. palmivora* colonization, and susceptibility to oomycete and AM colonization remains largely unknown. In mycorrhizal symbiosis, the *RAM2* compatibility gene involved in lipid metabolism is under the control of *RAM1* (Gobbato *et al.*, 2013; Bravo *et al.*, 2017). In contrast, *RAM2* controls surface entry by *P. palmivora* in a *RAM1*-independent manner (Rey *et al.*, 2015). It would therefore be relevant to characterize interactors of *RAD1* such as TF80 and TF124 (Park *et al.*, 2015) for their contribution in seedling elongation and oomycete compatibility to understand further whether and how RAD1 recruits different partners in order to direct such diverse signalling inputs.

In conclusion, we demonstrate that root interactions with a pathogen, the oomycete *P. palmivora*, involve a *Medicago* gene previously implicated specifically in root symbiosis with bacterial and filamentous microbes. Furthermore, our root length association study points to a pathogen-inducible modulation of seedling root length via *RAD1*. Our findings broaden the view of common principles of plant–microbe interactions and provide new options to breed for quantitative resistance against diseases caused by oomycete pathogens.

## Supplementary data

Supplementary data are available at *JXB* online.

Fig. S1. Time course of *P. palmivora* AJ-td infection in *M. truncatula* A17.

Fig. S2. Linkage disequilibrium (LD) analysis of the region encompassing *RAD1*.

Fig. S3. Genome-wide display of negative logarithms of the association *P*-value for all single nucleotide polymorphisms (SNPs).

Fig. S4. Root expression of *hpRAD1* does not affect overall development of composite plants.

Fig. S5. *Medicago truncatula* A17 roots expressing *hpRAD1* silencing constructs are not impaired in the defence response triggered by *P. palmivora* Lili-YKDEL.

Dataset S1. Total numbers and root length of each individual *P. palmivora*-infected and uninfected seedling for each accession in all repetitions and alleles present in each accession for the two SNPs significantly associated with mean root length

Table S1. List of primers used in this study, their applications, and genomic identifier of target sequences

supplementary Table S1

supplementary_figures_S1_S5

Supplemental Data S1
