# Supplementary material for: The Medicago truncatula GRAS protein RAD1 supports arbuscular mycorrhiza symbiosis and Phytophthora palmivora susceptibility
Source: J Exp Bot. 2017 Nov 25;68(21-22):5871–81. doi: 10.1093/jxb/erx398 (PMC5854134; doi:10.1093/jxb/erx398)
Supplement: supplementary_figures_S1_S5 [file erx398_suppl_supplementary_figures_s1_s5.pdf]

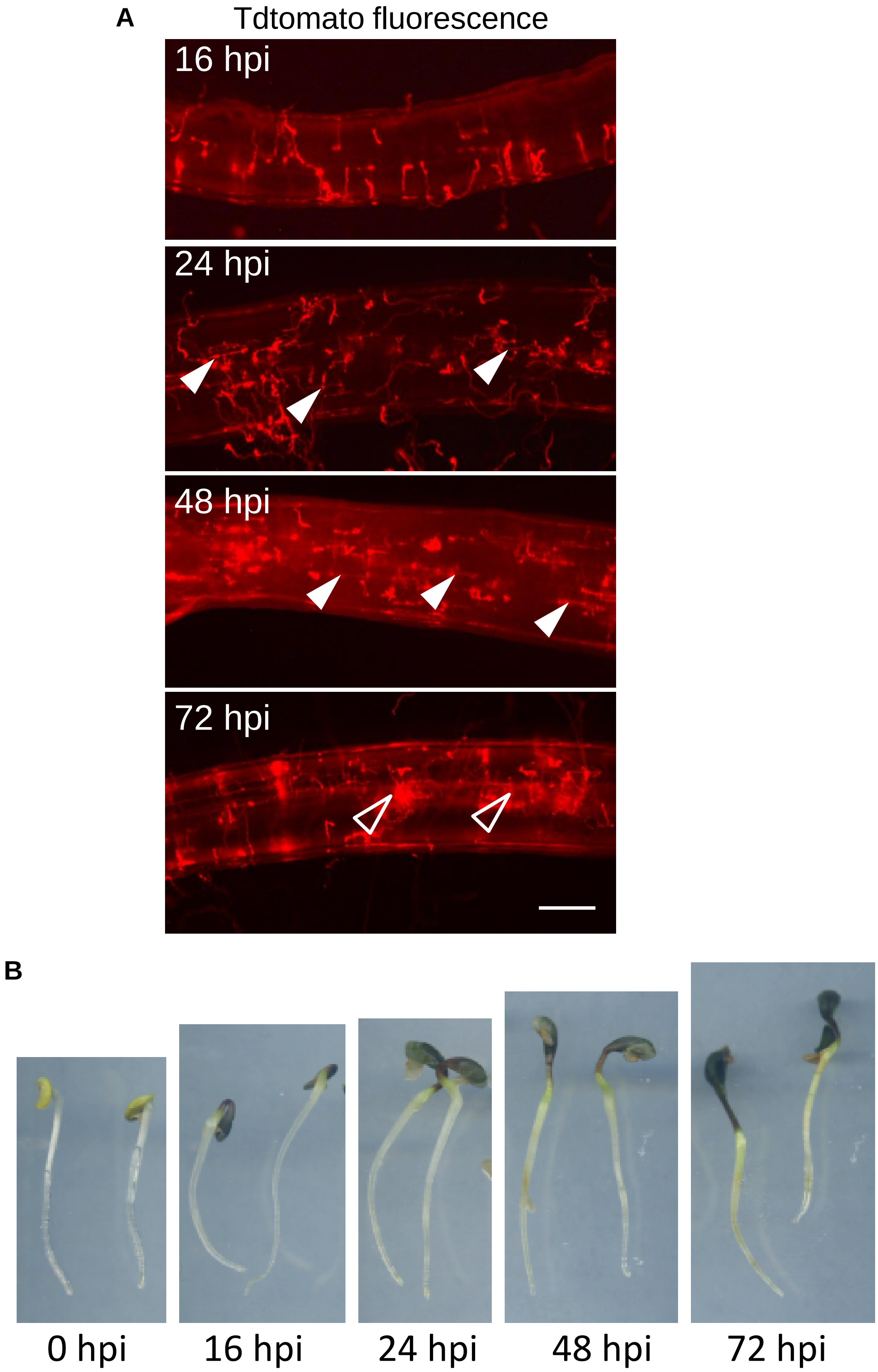

Supplementary Figure S1. Time course of *P. palmivora* AJ-td infection in *M. truncatula* A17. **(A)** microscopic assessment of colonisation. Filled arrowheads depict cortical biotrophic hyphae, empty arrow heads point to vascular colonisation (bar = 100µm). **(B)** Disease symptom development (root browing).

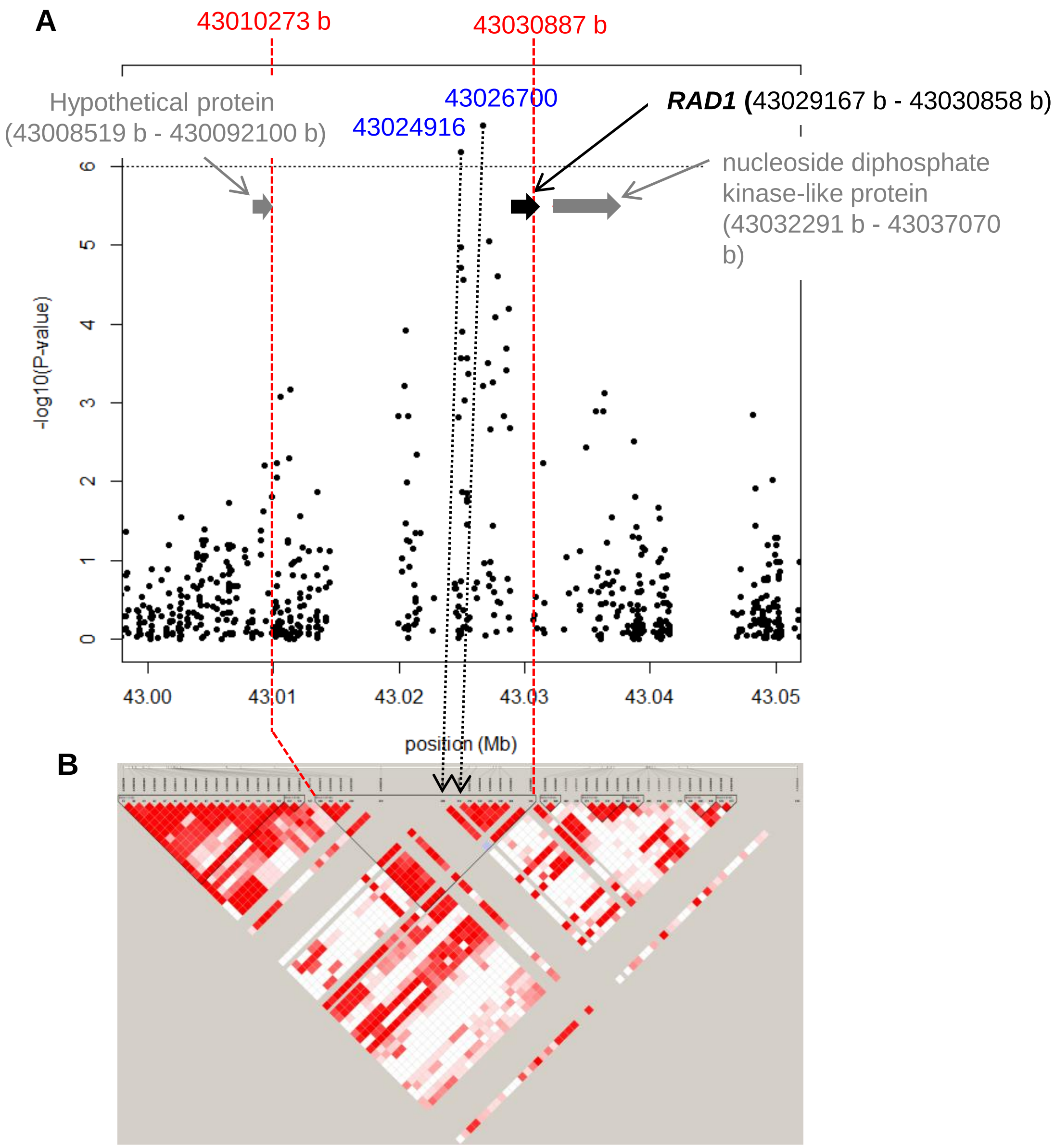

Supplementary Figure S2. Linkage disequilibrium (LD) analysis of the region encompassing *RAD1*. *RAD1* is the only gene within a LD block of ~ 20.6 kb. Red dashed lines in **(A)** limit a black triangle representing a block of LD in **(B)** where red squares show LD (D' estimator) between each SNPs. SNPs within the *RAD1*-containing LD block (including the blue highlighted two GWAM significant SNPs) are all in complete or nearly complete LD (D' =1). SNPs used for LD have MAF>0.20 and 90% genotype scoring.

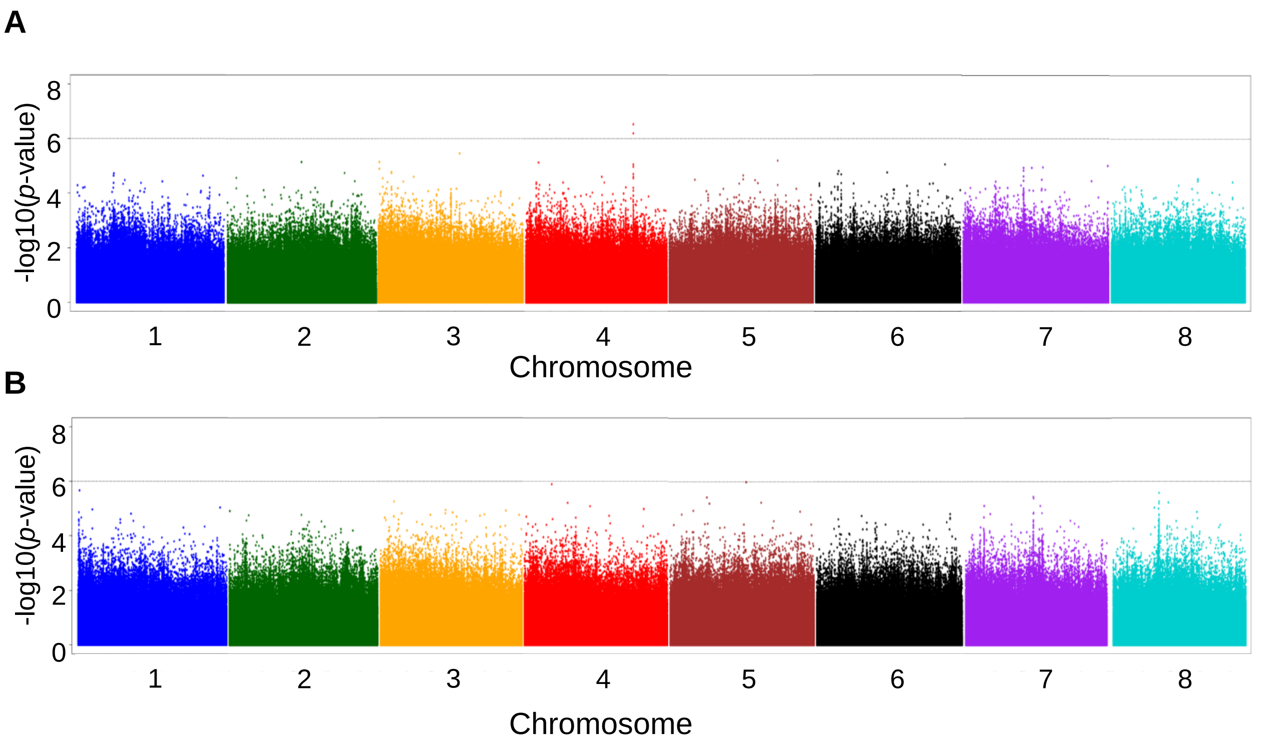

Supplementary Figure S3. Genome-wide association mapping of seedling length on infected and uninfected conditions. -log10(p-value) are displayed for all SNPs tested. **A** Two SNPs are above the significance threshold of –log10(6) in the association study for root length after *P. palmivora* infection; **B** No significantly associated SNPs were identified in the association study of seedling root length in the absence of an infection

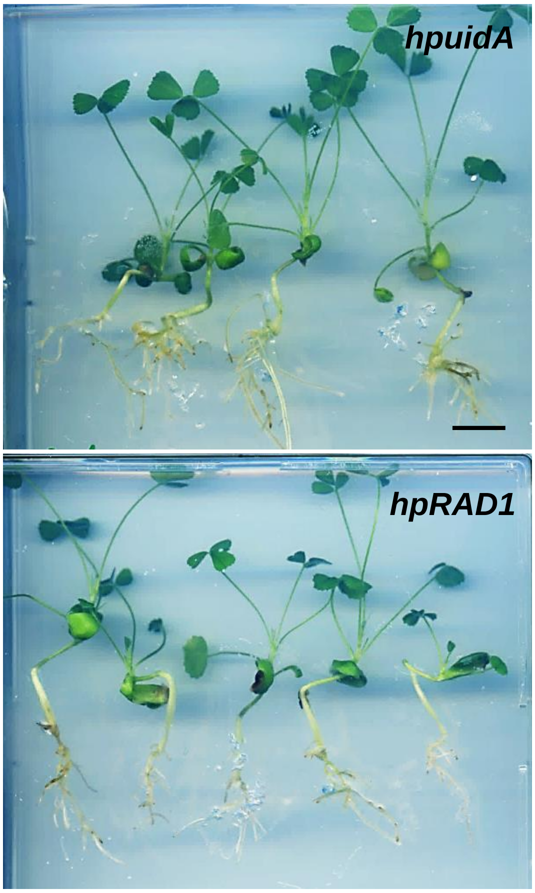

Supplementary Figure S4. Root expression of *hpRAD1* does not impact root habitus of composite plant. Composite plants expressing hairpin RNAi fragments in their roots grown for 6 weeks in sterile culture prior their inoculation with *P. palmivora* (bar = 1cm).

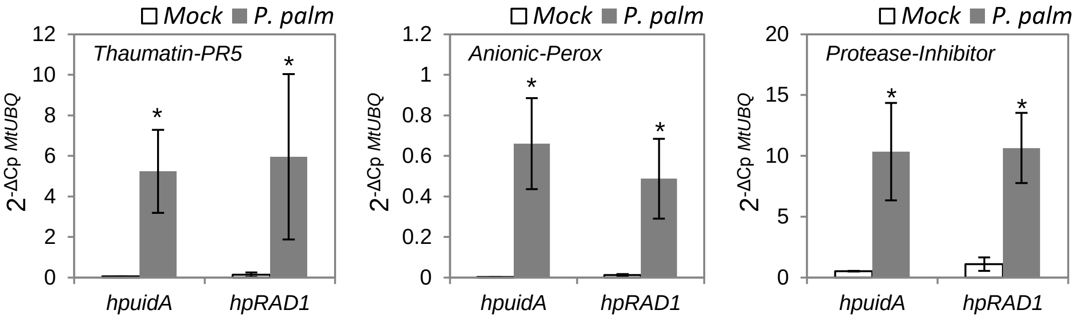

Figure S5. *M. truncatula* A17 roots expressing *hpRAD1* silencing constructs are not impaired in the defence response triggered by *P. palmivora* Lili-YKDEL. Transcript levels of *Thaumatin-PR5* (Mtr.9569.1.S1_at)*, Anionic Peroxidase* (Medtr4g083710) and *Protease Inhibitor* (Medtr1g075340) were quantified in control (Mock) conditions (white bars, n=3) or upon infection with *P. palmivora* Lili YKDEL (grey bars, n=4) of composite plants transformed with *hpuidA* or *hpRAD1* constructs. Student's *t*-test was applied to compare gene expression between conditions and between constructs. Defence marker gene expression was standardised using *MtUBQ* (Medtr3g091400) as housekeeping gene and the $2^{-\Delta Cp}$ method (*=p<0.05). Error bars show SEM.
